# Reimagining medical education: integrating medical humanism and narrative medicine into a new educational paradigm

**DOI:** 10.3389/fmed.2026.1761177

**Published:** 2026-01-16

**Authors:** Zhitao Hou, Jing Chen, Hongwei Guo

**Affiliations:** College of Basic Medical and Sciences, Heilongjiang University of Chinese Medicine, Harbin, Heilongjiang, China

**Keywords:** artificial intelligence, educational reform, ethical dilemmas, humanism, medical education

## Abstract

**Background:**

Medical education has increasingly prioritized technological competence, often at the expense of humanistic values central to patient-centered care.

**Aims:**

This Mini Review examines how medical humanism and Narrative Medicine can be systematically integrated into contemporary medical education to rebalance technical expertise and humanistic care.

**Key findings:**

Current evidence suggests that Narrative Medicine enhances empathy, communication skills, professional identity, and ethical sensitivity. However, integration efforts remain fragmented and are frequently constrained by curricular overload, insufficient faculty preparation, and misaligned assessment systems.

**Conclusion:**

Embedding Narrative Medicine within core curricula, supported by interdisciplinary collaboration, longitudinal programs, and rigorous evaluation frameworks, offers a feasible pathway toward a more holistic and sustainable medical education paradigm.

## 1 Introduction

Medical education has long sought to balance professional expertise with humanistic concer (1). From the Hippocratic principles of beneficence and non-maleficence in ancient Greece, through the Renaissance integration of medicine with philosophy and the arts, medical training has gradually expanded beyond technical instruction. It has evolved into a comprehensive educational framework that addresses patients’ physical, psychological, and social wellbeing (2). However, in a global medical landscape shaped by rapidly advancing technologies, the tension between scientific rigor and humanistic values has become increasingly pronounced (3, 4). As a result, medical humanities education often remains marginalized within curricula. Persistent challenges include limited curricular space, difficulties in quantitatively assessing humanistic competencies, and fragmented implementation in clinical training (5).

In recent years, changes in care models and educational thinking have led to the growing integration of Narrative Medicine into medical education systems (6). By listening to patients’ illness narratives and understanding their lived experiences, narrative medicine helps to strengthen healthcare professionals’ empathy, communication skills and clinical judgment (7). Evidence from empirical research and teaching practice indicates that Narrative Medicine, when used educationally, promotes the development of humanistic caring, narrative competence and professional identity among medical students and residents (8). Nevertheless, prior research points out that the burdens of clinical work and conventional, technology-centered educational models continue to impede the widespread and structured adoption of narrative practices (9). Thus, a central question for current medical education reforms is how to implement Narrative Medicine in a structured and regulative way so that it yields enduring, concrete gains in clinical care (6). Against this backdrop, this paper aims to explore in depth the pathways for integrating medical humanism and Narrative Medicine, and, drawing on the latest international research in medical education, to propose concrete reform strategies that support their coordinated development and the overall optimization of medical education systems (10) (Table 1).

**Table 1:** Integrated framework linking medical humanism, narrative medicine, and educational reform in contemporary medical education.

Major theme	Core concepts/key arguments	Mechanisms/educational pathways	Outcomes for medical learners/systems	Reference
Historical Foundations of Medical Humanism	1. Medicine historically balances technical practice with ethical–humanistic commitments; 2. Hippocratic ethos to Renaissance integration of art & medicine.	Ethical principles, whole-person perspective, philosophical grounding of patient care.	Establishes moral grounding, reinforces patient-centered care values.	(1, 2, 24, 25)
Tension Between Technology and Humanism	1. Rapid technological growth reduces curricular space for humanities; 2. Fragmentation of humanistic competencies.	Tech-oriented focus draws attention away from empathy, communication, ethical reflection.	Decreased empathy, weakened doctor–patient relationships, poor holistic competencies.	(3–5)
Shifts in Modern Medical Education	Education moving from basic sciences → integrated → competency-based → humanistic–technological fusion.	PBL, CBME, integrated humanities programs.	Improved reasoning, teamwork, ethics, professional identity.	(11–15)
Rationale for Narrative Medicine Integration	1. Narrative Medicine bridges sciences & humanities; 2. Centers patient stories in care.	Close reading, writing, reflective practice, narrative interviews.	Enhances empathy, communication, diagnostic insight, ethical awareness.	(6, 7, 16, 17)
Contemporary Challenges in Medical Humanities	1. Humanities courses often marginalized; 2. Lack of measurable assessment; 3. Insufficient integration.	Limited faculty expertise, curricular gaps, insufficient institutional support.	Students show low humanistic literacy and narrative competence.	(20–23)
Educational Value of Narrative Medicine	Strengthens narrative competence, enhances professional identity, improves empathy & communication.	Reflective writing, patient-story engagement, art-based learning, interpersonal dialog.	Better OSCE performance, improved ethical reasoning, enhanced clinician wellbeing.	(28, 30, 31, 33, 34)
Evidence on Narrative Competence & Clinical Skills	Narrative competence correlates with empathy and self-efficacy.	Structured training in narrative listening, reflective writing.	Students demonstrate higher communication scores, deeper reflection.	(32)
Simulation & Narrative Integration	Narrative Medicine + simulation supports holistic learning.	Scenario-based simulation, high-fidelity models, role-play.	Improves adaptability, communication, ethical decision-making.	(39, 40)
Digital & Hybrid Narrative Approaches	Online narrative forums, digital storytelling, virtual patients.	Telemedicine-based narrative encounters, EHR narratives.	Broadens access; prepares learners for digital healthcare ecosystems.	(42, 43)
Curriculum Integration Strategies	Humanities integrated as core, not elective.	Required modules on humanism, narrative care.	Strengthens cultural competence, reduces empathy decline.	(20, 36, 37)
Interdisciplinary Teaching Model	Collaboration across arts, humanities, and medicine.	Team-based teaching, joint seminars, art observation, literature analysis.	Improves interpretive skills, meaning-making, communication.	(21, 36)
Longitudinal Reflective Programs	Long-term, structured exposure yields deeper learning than short courses.	Cycles of observation → narrative → reflection → re-interpretation.	Sustained empathy and professional identity formation.	(37, 38)
Assessment and Evaluation Frameworks	Need for standardized metrics (KPI: narrative competence, empathy, communication).	Reflective journal rubrics, OSCE, multi-source assessment, longitudinal tracking.	Ensures educational quality and measurable learning outcomes.	(44–46)
Future-Oriented Paradigms (AI + Humanism)	AI, big data, precision medicine require stronger humanistic foundation.	Hybrid pedagogy merging tech literacy with ethical & narrative competence.	Physicians who adapt to technological systems without losing human touch.	(47, 48)
Overall Contribution of Integrated Model	Blending medical humanism + narrative medicine + pedagogical reform leads to balanced training.	Curriculum redesign, faculty development, digital tools, interdisciplinary networks.	Produces clinicians who are technically proficient and deeply humanistic.	(10, 35)

## 2 Methods

A narrative Mini Review approach was adopted. Literature was identified through searches of PubMed, Web of Science, and Scopus using combinations of the terms medical humanism, narrative medicine, medical humanities, and medical education. Priority was given to peer-reviewed articles published between 2000 and 2025, with emphasis on recent (2024–2025) studies. Relevant books and policy reports were also included. Articles were thematically analyzed to synthesize historical foundations, educational models, implementation challenges, and future directions.

## 3 Reforming medical education: historical background and contemporary practice

### 3.1 Key historical milestones: major shifts in medical education

The modern medical education system originated in the early twentieth century, when demands for standardized and systematic training shaped a professionalized structure that unified basic medical sciences with clinical medicine (11). However, the dominance of scientific and technological paradigms during this period resulted in a weakening of humanities education within medical curricula. Gradually, medical education underwent several rounds of evolution: from systematic basic science training (basic theory, anatomy, physiology, pathology) to the later incorporation of PBL (problem-based learning) and integrated curricula that introduced ethics and social medicine into medical training (12, 13). Later, competency-based medical education (CBME) placed increasing focus on developing integrated competencies, including communication, ethical judgment, and cross-disciplinary teamwork (14, 15). In recent years, shifts in global healthcare systems and medical paradigms have driven medical education toward an integrated model that connects basic sciences, clinical practice, the humanities and social sciences, and emerging technologies. In particular, recent global discourse in medical education highlights “patient-centered care,” “holistic wellbeing,” and “human-oriented healthcare” (2). Narrative Medicine, blending humanistic understanding with practical clinical engagement, has gained increasing recognition. Studies suggest that narrative medicine is not only a conceptual framework but also a pedagogically and clinically valuable tool that bridges medical science and humanistic care (16, 17).

### 3.2 Modern developments: the present landscape and challenges of medical education

As the goals of medicine shift from disease treatment to lifelong health maintenance, medical education now requires students not only to acquire solid biomedical knowledge but also to develop communication skills, ethical awareness, and humanistic compassion (18, 19). However, in many medical schools, medical humanities courses remain subordinate, with limited teaching hours, insufficient curricular structure, and weak links to clinical practice—resulting in significant gaps in students’ humanistic literacy and doctor–patient communication skills (20). In response to these issues, several medical schools have started incorporating Narrative Medicine and broader humanities content into formal medical education. Methods such as film analysis, literature, patient narratives, reflective writing, and art-based observation are being adopted to present patients as whole individuals situated within emotional and social contexts (21). Meanwhile, some studies and educational programs combine narrative medicine with simulation teaching and scenario-based simulation to enhance students’ ethical reasoning, communication skills, and clinical adaptability (22). However, these efforts still face structural challenges, including insufficient institutionalization, lack of systematization, and incomplete evaluation mechanisms. Numerous studies indicate that the field still lacks standardized and quantifiable instruments for evaluating outcomes in Narrative Medicine education (23). In addition, in resource-limited regions or in medical schools with heavy curricular burdens, the integration of humanities courses is often overlooked (20). In summary, while medical education reform has begun to balance humanism with technology, achieving a truly systematic and structured integration of humanities and medical training requires sustained progress in pedagogical philosophy, curriculum design, faculty development, evaluation systems, and cultural adaptation.

## 4 Origins and evolution of medical humanism

### 4.1 Origins and core values of humanistic medicine

The roots of medical humanism extend to ancient Greek civilization. In the Hippocratic era, foundational principles of medical ethics emerged, and ideas such as “respect for life” and “honest protection of the patient’s interest” laid the groundwork for modern clinical professionalism (1). As time progressed, the Renaissance brought deep integration between medicine and fields such as art and philosophy; anatomical illustration, medical drawing, and the fusion of dissection with artistic observation collectively contributed to a humanistic shift in anatomy and medical practice (24). Meanwhile, Eastern traditional medicine (such as Chinese medicine) embodies deeply humanistic concepts: holism, unity of body and mind, harmony between humans and nature, and ethical principles such as “benevolence of the healer” and “medicine as an act of humanity” (25). These ideas align closely in spirit with the principles of modern medical humanism. Since the twentieth century, rapid advances in medical science and technology led to a period in which medical humanism was marginalized. However, with the shift toward the “biopsychosocial” model, humanistic care and medical ethics regained importance (26). Many reformers and scholars in medical ethics (e.g., Pellegrino) assert that medicine is both a science and an art, embodying a profound commitment to life and to the human condition.

### 4.2 Modern medical humanism: its evolution and mission

The goal of modern medical humanistic education is no longer limited to treating disease alone but to providing holistic care for patients as whole persons (2). Consequently, educational reform focuses not only on diagnostic and therapeutic skills but also on cultivating communication abilities, ethical awareness, cultural sensitivity, social responsibility, and professional identity. Emerging research indicates that incorporating humanities (literature, ethics, sociology, the arts) into medical curricula not only improves empathy, observational capacity, and cultural awareness but also reinforces ethical discernment, professional identity formation, and commitment to person-centered care (27). In this context, Narrative Medicine developed as a methodological and pedagogical approach. Through storytelling and attentive listening, reflective writing, art observation, patient narratives, and interdisciplinary dialog, it integrates humanistic spirit with clinical knowledge, adding warmth and depth to medical education (16, 28). Therefore, the central mission of modern medical humanistic education is to guide medicine back to its human-centered foundation: not only treating disease but understanding, respecting, and responding to the full spectrum of human needs—physical, psychological, social, and cultural (29).

## 5 The emergence and evolution of narrative medicine

### 5.1 Global perspectives and modern educational practices

Narrative Medicine was first proposed by Rita Charon as a bridge linking medical science with humanistic care (16). It emphasizes placing the “narratives” of patients, caregivers, healthcare professionals, and community members at the center of medical practice and education. In recent years, narrative medicine has been increasingly applied in medical education and clinical practice. Studies from 2023 to 2025 demonstrate that narrative medicine practices—such as reflective writing, patient narratives, artistic and cultural engagement, and digital storytelling—significantly improve empathy, narrative competence, communication skills, professional identity, and ethical awareness in medical learners (7, 30, 31). For example, a 2023 cross-sectional study involving 434 senior medical students at a Chinese medical university showed that narrative competence was significantly positively correlated with self-efficacy and empathy (*r* = 0.345, 0.492; *p* < 0.01) (32). The study further indicated that students’ overall narrative competence remained low, and that current higher education institutions lacked systematic training in narrative skills. Moreover, attempts to integrate narrative medicine with scenario simulation and high-fidelity simulation are emerging. This trend indicates that narrative medicine has shifted from a peripheral concept to a mainstream educational approach, improving relationships between clinicians and patients and potentially promoting a more humanistic healthcare system.

### 5.2 The value and limitations of narrative medicine in medical humanities education

The core of narrative medicine lies in listening to and telling patients’ stories, enabling clinicians to understand more fully how illness affects individuals and families, thereby improving communication, reducing disputes, and enhancing patient experience and safety. It calls for cultivating a “narrative self”—via reflection, writing, artistic practice, and dialog—so that clinicians become listeners, witnesses, and participants, not simply providers of technical procedures. Numerous studies have confirmed that narrative medicine interventions enhance empathy, professional identity, communication skills, ethical sensitivity, and humanistic literacy in medical students and residents (28, 30, 33). For example, research on obstetrics residents trained with case-based Narrative Medicine demonstrated superior OSCE scores, stronger narrative competence, better communication skills, and deeper reflective thinking compared with those taught conventionally (34). Another nationwide study of medical interns highlighted that narrative competence is a key contributor to their empathy and self-efficacy (32). However, current narrative medicine education faces several limitations: interventions are mostly small-scale and short-term, and research on long-term outcomes remains limited (30). Studies examining long-term effects—on care quality, doctor–patient relationships, burnout, and patient safety—are still lacking. In addition, many medical schools lack systematic curriculum design, faculty training, interdisciplinary teams across humanities and clinical fields, and standardized quantitative assessment tools. Therefore, effectively integrating narrative medicine into modern medical education requires comprehensive planning involving institutionalization, systematization, interdisciplinarity, and evaluability.

## 6 Integrated innovation in medical education: the synergy of medical humanism, narrative medicine, and pedagogical reform

**Figure 1:**
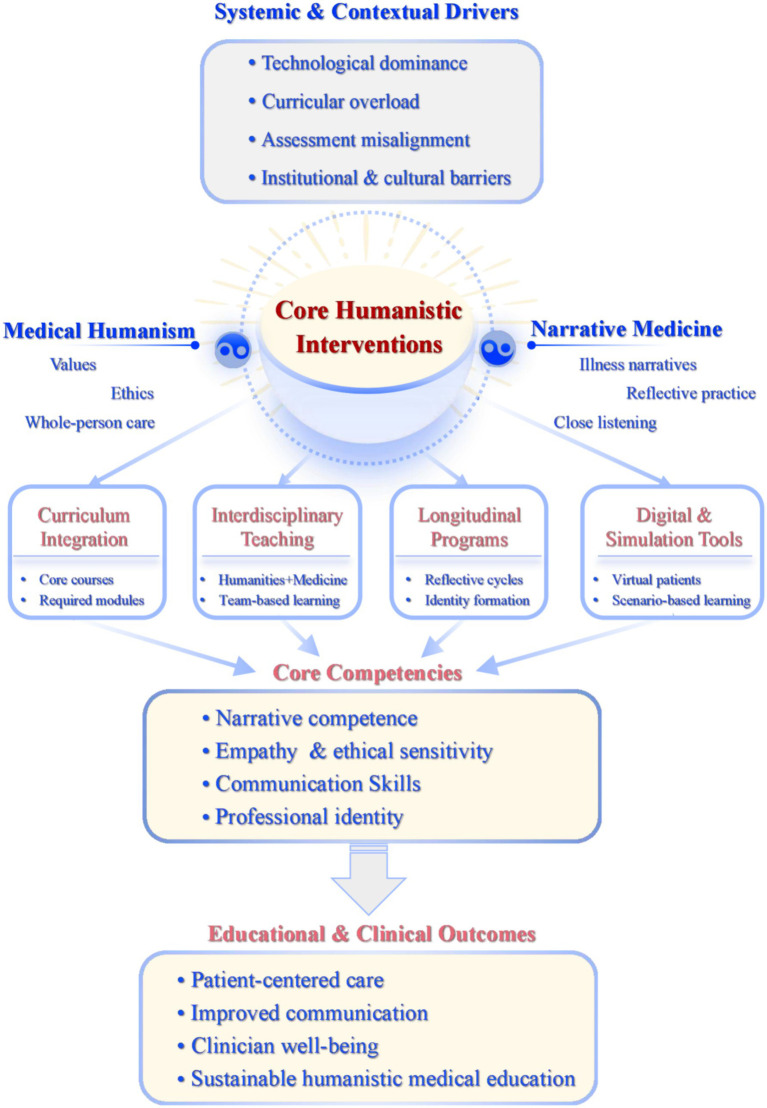
An integrative framework for embedding medical humanism and narrative medicine in contemporary medical education. This conceptual framework illustrates how medical humanism and narrative medicine can be systematically integrated into contemporary medical education to rebalance technological competence and humanistic care. Systemic drivers such as technological dominance, curricular overload, assessment misalignment, and institutional culture shape the educational context. Narrative medicine and medical humanism function as core humanistic interventions, which are operationalized through pedagogical pathways including curriculum integration, interdisciplinary teaching, longitudinal reflective programs, and simulation- and digitally-enhanced learning. These pathways foster key competencies—empathy, narrative competence, ethical sensitivity, and professional identity formation—ultimately contributing to patient-centered care, clinician wellbeing, and a more sustainable and humanistic medical education paradigm.

As medical education advances toward comprehensive interdisciplinary and transdisciplinary integration, medical humanism, Narrative Medicine, and educational reforms have become more closely interwoven. Narrative Medicine, as a key bridge connecting the humanities with medicine and linking theory with practice, introduces new pedagogical models and reform pathways to medical education (35). To synthesize the relationships among systemic challenges, pedagogical strategies, and educational outcomes, an integrative conceptual framework is presented in Figure 1, illustrating how medical humanism and Narrative Medicine can be operationalized within contemporary medical education (Figure 1).

### 6.1 Innovations in teaching models: curriculum integration, interdisciplinarity, and longitudinal programs

First, in terms of curriculum integration, incorporating Narrative Medicine and medical humanities into the core medical curriculum—rather than treating them as elective or peripheral modules—has become a major trend in contemporary educational reform. For example, a module such as “Narrative Medicine and Humanistic Care” may be added alongside basic science and clinical curricula, allowing literature, ethics, sociology, cultural studies, and art observation to be integrated systematically into medical education (36). Several institutions have developed Narrative Medicine into multi-year, interdisciplinary educational programs, demonstrating the transition of medical education toward more structured and enduring humanistic training (6, 37). Second, interdisciplinary teaching integrates knowledge from medicine, literature, sociology, psychology, the arts, and anthropology, and utilizes diverse methods such as team teaching, joint seminars, clinical case analysis, reflective writing, art observation, theatre simulation, and film/literature critique to help students understand disease and patient narratives from multiple perspectives, fostering meaning-making and clinical empathy (20, 36). Moreover, compared with short-term lectures or isolated teaching activities, longitudinal and reflective programs are more conducive to the sustained development of narrative competence, empathy, ethical sensitivity, and professional identity (38). Such programs are typically characterized by structured, continuous, and deeply reflective learning, allowing students to deepen their understanding of patient experience and humanistic care through cycles of real narratives, clinical observation, and reflective writing. Overall, these innovations collectively shift medical education from purely technical training toward a more integrated model that balances technology and humanism, making medical talent development better aligned with the holistic, complex, and human-centered nature of future medicine.

### 6.2 Innovations in pedagogical methods and tools: diversity, simulation, and digital approaches

First, case-based and simulation-based methods: as previously noted, integrating Narrative Medicine with high-fidelity simulation not only trains students in clinical skills but also encourages them to attend to patients’ holistic, ethical, and communicative needs, making it a highly promising teaching strategy (39, 40). Second, reflective writing and storytelling: encouraging students to express and reflect through writing, patient narratives, family stories, and cultural or artistic media (such as literature, painting, and theatre) constitutes a central component of Narrative Medicine (41). Third, digital and hybrid approaches: as digital health and telemedicine evolve, Narrative Medicine may incorporate digital platforms such as online discussion forums, narrative components in EHRs, virtual/synthetic patient simulations, and narrative-based telemedicine encounters (42, 43). These approaches not only expand the reach of Narrative Medicine education but also align with the evolving landscape of modern healthcare.

### 6.3 Evaluation and quality assurance: building a rigorous assessment framework

The expansion of Narrative Medicine education must also address the crucial question of how to assess its effectiveness and value. Existing literature has called for the development of standardized, reliable, and quantifiable key performance indicators (KPIs) to measure domains such as narrative competence, empathy, communication skills, professional identity, and ethical sensitivity (16, 44). In practice, validated instruments such as structured reflective writing rubrics (e.g., the REFLECT rubric), empathy scales (e.g., the Jefferson Scale of Empathy), and professionalism- or communication-focused OSCE stations have been proposed to operationalize these domains. Educational reform should incorporate the development of evaluation mechanisms—such as reflective journals, OSCEs, patient/peer/faculty assessments, multidimensional questionnaires, and longitudinal follow-up—to ensure that Narrative Medicine education moves beyond conceptual ideals and is translated into genuine humanistic literacy and clinical competence for medical students and physicians (44–46).

### 6.4 Toward the future: a medical education paradigm that integrates technology, humanism, and social dimensions

With the rapid development of AI, big data, telemedicine, and precision medicine, medical education needs to foreground a holistic perspective that synthesizes technological, humanistic, and social components (47, 48). Narrative Medicine and medical humanities provide both the theoretical foundation and practical pathways for such an integrative perspective. Moving forward, medical education must safeguard clinical and scientific competencies while incorporating narrative pedagogy, arts and humanities, interdisciplinary collaboration, ethics instruction, cultural sensitivity training, and sociological/public health perspectives, thereby shaping physicians who can truly understand patients, honor individual dignity, care for society, and thrive in the healthcare systems of the future (10).

## 7 Discussion: critical reflections, barriers, and future directions

Although the integration of medical humanism and Narrative Medicine has gained growing conceptual and empirical support, real-world implementation within medical education systems has often remained limited (49). A critical examination of prior integration attempts suggests that these challenges stem less from theoretical inadequacy than from structural, cultural, and institutional constraints (50).

One major barrier is curricular overcrowding. Medical curricula are densely packed with biomedical and technical content, leaving limited protected time for sustained humanistic training. As a result, Narrative Medicine is frequently introduced as a short-term, elective, or extracurricular activity, which restricts its continuity and educational impact. Without longitudinal integration, narrative-based interventions risk remaining symbolic rather than transformative.

A second challenge lies in insufficient faculty preparation and institutional support. Many medical educators lack formal training in narrative pedagogy, reflective facilitation, or humanities-based assessment (50). Consequently, Narrative Medicine may be reduced to isolated reflective writing exercises without structured guidance or feedback. Moreover, in technology-driven academic environments, humanistic teaching is rarely prioritized in faculty evaluation or promotion systems, further limiting sustainable implementation.

Third, the absence of standardized and validated assessment frameworks has constrained scalability. Although outcomes such as empathy, narrative competence, and professional identity are widely acknowledged as essential, they are often perceived as difficult to measure (51). This perception contributes to skepticism among curriculum committees and administrators, particularly in comparison with competency-based and examination-oriented training models.

Importantly, Narrative Medicine should not be positioned as competing with scientific or technical education. Rather, emerging evidence suggests that narrative competence complements clinical reasoning, ethical judgment, and communication, especially in complex and emotionally charged clinical encounters (51). Framing Narrative Medicine as an integrative competency—rather than an additional curricular burden—may enhance its feasibility and acceptance.

Future progress will require system-level alignment, including protected curricular time, interdisciplinary faculty development, institutionally endorsed learning outcomes, and longitudinal assessment strategies (50). In addition, future research should prioritize longitudinal and mixed-methods studies to examine the sustained effects of narrative training on clinical performance, physician well-being, patient outcomes, and healthcare culture.

## 8 Conclusion

This paper systematically reviews and synthesizes the developmental trajectory of medical humanities and Narrative Medicine, and, drawing on recent advances in medical education and Narrative Medicine research, elucidates the importance of deeply integrating the two within modern medical education systems. Through empathy, reflection, narrative understanding, and cultural sensitivity, Narrative Medicine infuses humanistic spirit into medical education and serves as a vital pathway for achieving balance between technical competence and humanistic care.

Although challenges remain in curriculum design, faculty development, teaching resources, and evaluation mechanisms, progress can be made through curriculum integration, interdisciplinary teaching, longitudinal reflective programs, innovation in teaching methods and tools, and the establishment of rigorous quantitative assessment systems—ultimately enabling Narrative Medicine and medical humanities to become central pillars of medical education. Future medical education should aim to cultivate healthcare professionals who are not only clinically competent but also endowed with humanistic care and social responsibility, enabling them to address the complex challenges of 21st-century medicine, public health, and societal well-being.

This study calls on medical schools worldwide, healthcare systems, and education policymakers to work together in advancing this new paradigm of medical education, ultimately achieving truly patient-centered healthcare and medical training.
